# Sustainable Overproduction of the Bacterial Pigment Indigoidine as a Natural Colorant: Stability Evaluation, Inhibition of Obesity‐Diabetes‐Related Digestive Enzymes, and Activation of AMPK–AKT

**DOI:** 10.1002/mbo3.70429

**Published:** 2026-10-07

**Authors:** Tahani Ali Asseri

**Affiliations:** ^1^ Department of Biology, College of Science King Khalid University Abha Saudi Arabia

**Keywords:** AKT, AMPK, bioavailability, glycolysis, indigoidine, optimization

## Abstract

The increased focus on bacterial pigments can be attributed to their greater safety and eco‐friendliness when compared to artificial food colorants, which can lead to severe health problems. This research was designed to optimize IND production at varying growth conditions, to assess its chemical stability, and to study its possible biological activities associated with diabetes, obesity, and insulin resistance. Medium types played an important role in both BL21‐DE3 cell growth and IND production. The BL21‐DE3 cell growth was maximum in PGB medium (2.76 g/L/24 h), then in LB‐O medium (1.29 g/L/24 h), LB medium (1.14 g/L/24 h), and NB medium (0.94 g/L/24 h). The IND production was 75.8, 49.8, and 38.9 mg/L/24 h in LB, NB, and PGB media, respectively, while the experimental maximum IND production was achieved in LB‐O medium (177.4 mg/L/24 h), which closely resembled the theoretical IND production of 180 mg/L/24 h. IND exhibited high cellular absorption properties, low cytotoxicity, and dose‐dependent inhibition of α‐amylase and lipase enzymes at IC_50_ values of 0.69 and 0.37 µg/mL, respectively. Furthermore, IND enhanced 5′‐adenosine monophosphate‐activated protein kinase (AMPK) and protein kinase B (AKT) signaling pathways at EC_50_ values of 1.36 and 4.59 µg/mL, respectively. The binding affinity of IND was also observed towards α‐amylase, lipase, AMPK, and AKT enzymes via molecular docking with binding energies (BE) of −7.4, −8.1, −6.9, and −5.5 kcal/mol, respectively. All these findings were confirmed through human spermatozoa culture, where IND stimulated glucose metabolism and AMPK and AKT signaling pathways.

## Graphical abstract

Production of indigoidine (IND) increased from 1.04 to 2.76 g/L using an optimized LB medium composed of glucose, l‐glutamine, tryptone, yeast extract, MgSO_4_·7H_2_O, and KH_2_PO_4_. The IND has significant inhibition properties against amylase and lipase enzymes, stimulates AMPK and AKT pathways, and improves glucose uptake. With its high stability, low toxicity, and powerful physiological activities, it is suggested to be useful for food coloring and ecotherapy against metabolic disorders.

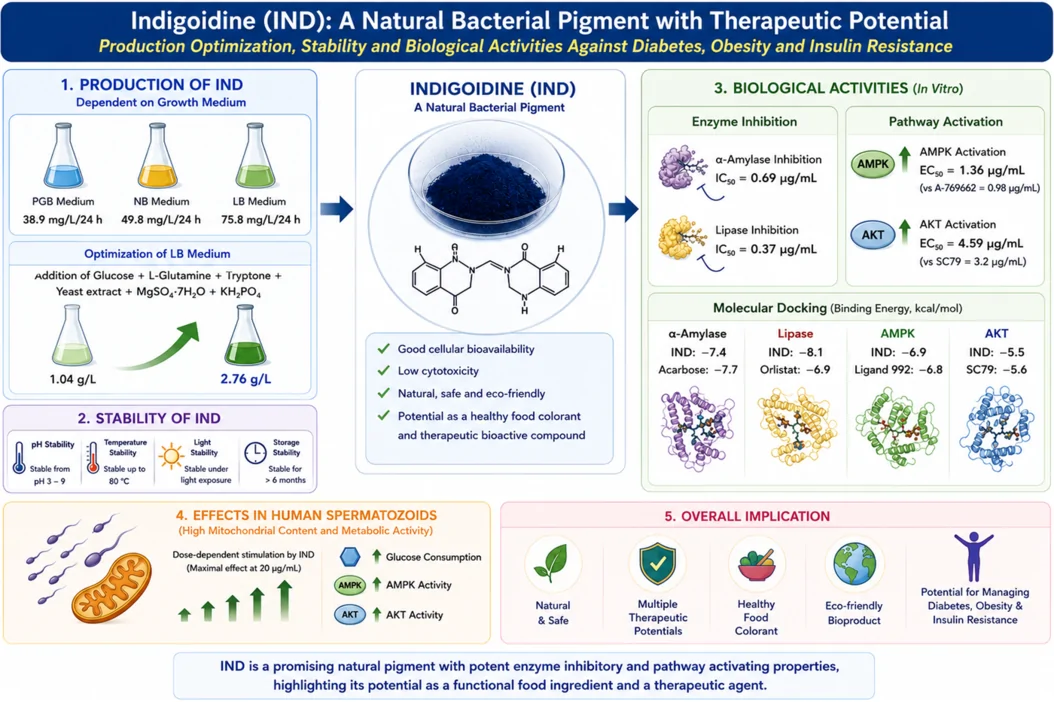

## 1 Introduction

Various studies have shown that synthetic colorants, which are extensively used in foods, pharmaceuticals, and cosmetics due to their low cost and high stability, may cause significant risks to human health and the environment, raising growing concerns worldwide (Abdella, Alanazia, Alreshidi, Al‐Enazy, et al. 2025; Abdella, Alreshidi, et al. 2025; Chaubal et al. 2026). It has been reported that several commonly used dyes, such as Allura Red, Tartrazine, Sunset Yellow, and Ponceau can induce various toxic effects in consumers, such as oxidative damage in different organs, systemic inflammation, immune system suppression, decline in male fertility, and several other physiological disorders (Abdella, Alanazia, Alreshidi, Al‐Enazy, et al. 2025; Abdella, Alreshidi, et al. 2025; Chaubal et al. 2026). Furthermore, these colorants can cause allergic reactions that may affect the skin, respiratory system and the digestive tract, especially in children (Aldabayan 2025; Feketea and Tsabouri 2017). From an environmental perspective, the high stability and poor biodegradability of synthetic dyes promote their persistence and accumulation in water, soil, plants, and animals, leading to ecosystem disruption, while their environmental transformation may generate toxic and potentially carcinogenic by‐products that pose significant risks to both environmental and human health (Kumari 2024; Vats et al. 2022).

To avoid the harmful effects of toxic chemical colorants on public health, the environment, and food quality, it is crucial to limit and reduce the excessive use of synthetic dyes while developing natural, eco‐friendly, sustainable, and environmentally responsible alternatives. Among these options are the use of natural pigments from microalgae like C‐phycocyanin (AlRashidi et al. 2026 Abdella, et al. 2026), from lichens like parietin (Abdella, Alanazia, Alreshidi, Al‐Enazy, et al. 2025), plants rich in betalains (El‐Sofany et al. 2025a), and there are also bacterial natural pigments, which include prodigiosin and violacein (Abdella, Alardan, et al. 2025; El‐Sofany et al. 2025). These natural pigments, in addition to being regarded as more responsible and healthier colorants, can be classified as highly bioactive compounds with therapeutic potential. They are defined by their renewability, compatibility with biological structures, and growing importance in the food, pharmaceutical, and cosmetics sectors (Ali et al. 2026; Garcia‐Gonzalez et al. 2014; Horta et al. 2019; Sebola et al. 2019). Bacteria are one of the best eco‐friendly resources that have shown significant capacities of sustainable high‐production yields of nontoxic natural pigments, along with other therapeutic effects, such as anti‐inflammatory, anti‐allergic, anti‐neoplastic, anti‐cancer, and antioxidant effects (Hassan et al. 2025; Kesbiç and Gültepe 2022; Mahrouk et al. 2025; Shekh et al. 2026; Youssef 2026). Consequently, bacterial pigments have attracted considerable attention for applications in the textile, food, cosmetic, and pharmaceutical sectors. Among bacterial pigments, indigoidine (IND), a naturally occurring blue pigment derived from many bacteria, has been widely studied because of its exciting pharmacological effects. Many reports have emphasized that IND shows remarkable antioxidant activity, which allows the pigment to remove reactive oxygen species and decrease oxidative stress associated with different chronic diseases (Panchanawaporn et al. 2022). It is also known to show antimicrobial activity toward several disease‐causing microorganisms (Kong et al. 2019), indicating a protective role in microbial interaction. In addition, IIND can display anti‐inflammatory activity via modulation of inflammatory agents and cellular redox balance. Since it is a natural compound with low toxicity, IND is expected to be promising for pharmaceutical use and other biomedical purposes (Kong et al. 2019). Given the importance of bacterial pigments in food and pharmacological applications, the optimization of fermentation, isolation, and stabilization processes represents a major industrial challenge, with the aim of increasing the production yields of these pigments for various applications. One of the key approaches is the optimization of fermentation conditions as well as culture medium composition, which constitutes an effective strategy to enhance the microbial productivity of bioactive and nontoxic pigments. In this context, the objective of our study is, first, the selection of an appropriate culture medium for improved IND production. Second, we perform nutritional and physicochemical optimization of the selected medium using experimental assays and mixture design approaches, followed by the evaluation of production yield, cellular stability, and toxicity. Finally, we assess for the first time the effect of IND on key enzymes involved in obesity, such as lipase, in diabetes, such as alpha‐amylase, and in insulin resistance through 5′‐adenosine monophosphate‐activated protein kinase (AMPK) and protein kinase B (AKT) pathways.

## 2 Materials and Methods

### 2.1 Production of Crude IND

The bacterial strain used was a nonpathogenic recombinant *Escherichia coli* BL21‐DE3 (competent cells, Novagen, ref. 69450‐4), prepared from 18‐h cultures in mid‐exponential growth phase. Precultures were grown in 250 mL Erlenmeyer flasks containing 50 mL of Luria–Bertani (LB) medium (tryptone 10 g/L, yeast extract 5 g/L, NaCl 10 g/L, pH 7.0) at 30°C with shaking at 180 rpm. For initial screening, IND production was evaluated in several media, including Nutrient Broth (NB) (peptone 10 g/L, NaCl 5 g/L, beef extract 3 g/L), Tryptic Soy Broth (TSB) (casein peptone 17 g/L, soy peptone 3 g/L, glucose 2.5 g/L, NaCl 5 g/L, K_2_HPO_4_ 2.5 g/L), and Peptone Glycerol Broth (PGB) (meat extract 10 g/L, peptone 10 g/L, glycerol 10% v/v). The pH of all media was adjusted to 7.0 ± 0.2. Cultures were inoculated with 10% (v/v) bacterial inoculum and incubated under shaking at 180 rpm at various temperatures. Samples were collected at 0, 15, 18, 20, 24, 30, and 36 h to measure cell density (OD_600_), pH, and IND production. To induce IND production, the concentrations of several nutrients such as tryptone, yeast extract, and NaCl were optimized, along with the addition of other components including glucose, l‐glutamine, and mineral salts, which are known for their potential inductive effects on IND production. In addition, the physical and chemical conditions during incubation, such as temperature, pH, and agitation were similarly altered. The alterations were done via an experimental process employing a mixture design technique, and all experiments were done thrice for validation purposes.

### 2.2 Preoptimization of Nutritional and Physicochemical Factors in LB Medium

First, after identifying LB as the most productive medium for IND production, although not the one supporting the fastest bacterial growth, we proceeded to optimize the selected LB‐based culture medium in terms of nutrient composition and incubation conditions. This optimization was carried out using statistical analysis methods based on a Plackett–Burman design. This mixture design helped us identify the key factors affecting the rate of IND production through targeted and guided experimental assays. Using this software, we were able to investigate the most influential parameters affecting IND production, including physical, chemical, and nutritional factors (medium composition and environmental conditions). The variables tested included tryptone (0.8%–1.75%), yeast extract (0.3%–0.95%), NaCl (0.3%–1.25%), as well as the addition of new nutrients such as glucose (0.25%–1.25%), MgSO_4_·7H_2_O (0.012%–0.075%), KH_2_PO_4_ (0.0025%–0.175%), and l‐glutamine (0.25%–2.5%), along with initial biomass concentration ranging from 1.8 to 2.7 g/L. In addition, several incubation parameters were also optimized, including temperature (28°C–30.5°C), pH (6–8.3), incubation time (15–36 h), and agitation speed (160–210 rpm) El‐Sofany et al. 2025; Ali et al. 2026.

### 2.3 Final Optimization of LB Medium for Enhanced IND Production

After a first optimization step, a final optimization was performed to theoretically determine the optimal conditions using a statistical optimization approach based on a central composite design (CCD), implemented with Minitab statistical software (Minitab® 19, Minitab LLC, State College, PA, USA). In this step, only three key variables were further optimized tryptophan, glucose, and yeast extract concentrations while all other parameters were kept constant. The response variables evaluated were IND productivity and cell density, allowing simultaneous assessment of pigment production efficiency and bacterial growth.

### 2.4 IND Extraction

IND was isolated using the method described by Zhang et al. (2014). The protocol involves harvesting bacterial cells by centrifugation of the culture broth at 6000 × g for 10 min at 4°C. The cells harvested from the optimized LB medium after 24 h of incubation were resuspended in phosphate buffer at a ratio of 5 mL of buffer per 1 g of biomass of IND‐producing bacteria. The phosphate buffer contained 50 mM sodium phosphate (NaH_2_PO_4_/Na_2_HPO_4_) at pH 7.0. Sonication was performed using a sonicator operating at a frequency of 25 kHz and at 40%–60% of its maximum power. Sonication was performed in pulses of 5–10 s with 10 s intervals between pulses, for a total duration of 5 min, to lyse the cells and release IND. After sonication, the mixture was centrifuged again at 10,000 × g for 15 min at 4°C. The supernatant was then filtered through a 0.22 µm membrane to remove residual cell debris. The resulting filtrate containing IND was concentrated and subsequently lyophilized to obtain a dry powder. The final product was stored at −20°C in the dark until further use.

### 2.5 Evaluation of IND Purity

The isolated IND was homogenized to a final concentration of 5 mg/mL in DMSO. The purity of the bacterial pigment was initially determined by UV–Visible spectrophotometry at its maximum absorbance (λmax = 600 nm; OD = 0.90). This purity was further confirmed by LC–MS using an Agilent single quadrupole mass spectrometer equipped with an electrospray ionization (ESI) source, coupled to an Agilent 1260 HPLC system comprising a G1311 quaternary pump, a G1322 degasser, and a G1315 diode array detector (DAD) (Agilent Technologies, Santa Clara, CA, USA). A gradient elution was performed from 10% methanol in water to 90% methanol in water over 25 min using an Agilent Eclipse XDB‐C18 RP‐HPLC column (4.6 × 150 mm, 5 µm), at a flow rate of 1 mL/min, in accordance with previously reported by (Yu et al. 2013; Gromek et al. 2016).

### 2.6 Evaluation of IND Stability

To evaluate the stability of IND, the pigment was dissolved in DMSO at a concentration of 100 µg/mL. Aliquots of 10 mL were diluted in buffer systems covering a pH range from 1 to 12, using citrate buffer for acidic conditions, phosphate buffer for neutral conditions, and NaOH‐adjusted buffer for alkaline conditions, while maintaining constant ionic strength by adding neutral salts. The stability of IND was evaluated under different conditions, including pH ranging from 1 to 12, storage duration at room temperature in both dark conditions, and exposure to light and UV radiation. Thermal stability studies were conducted by incubating the IND solution at temperatures ranging from 4°C to 100°C for 48 h. Photostability assessments were performed by exposing samples to normal light or storing them in complete darkness under identical conditions. After each treatment, the remaining IND content was quantified using DAD‐HPLC analysis. The half‐life (t½) was determined based on the time required for a 50% loss of pigment stability.

### 2.7 Evaluation of IND Toxicity on Human Erythrocytes

The cytotoxicity of IND was evaluated using a hemolysis assay on human erythrocytes to assess its effect on membrane integrity, as described by Cardoso et al. (2019). His assay was conducted using freshly isolated erythrocytes from the peripheral blood of a healthy 25‐year‐old volunteer, after obtaining informed consent and approval from the National Ethics Committee (TNAPO‐06‐B‐071; approval number TNC#2025‐3125), collected in EDTA‐containing tubes. After three washes with PBS buffer (0.01 M, pH 7.4), the erythrocytes were cultured in PBS supplemented with 10 mmol/L glucose as a glycolytic substrate, together with penicillin G (300 mg/L) and streptomycin (500 mg/L) as antimicrobial agents. Hemolytic activity, determined spectrophotometrically at 546 nm for the quantification of hemoglobin levels, was measured using a commercial kit from Biolabo (France) (reference 3502200).

### 2.8 Evaluation of the Membrane Bioavailability of IND

Equal aliquots of erythrocytes suspension were added into sterile Erlenmeyer flasks and exposed to different concentrations of IND (0.5–50 µg/mL) for 4 h at 37°C under slight stirring according to standard hemolysis procedures that prevent uncontrolled erythrocytes membranes breakdown. Afterwards, the samples were centrifuged at 1800 rpm for 10 min, which allows to isolate extracellular and cellular fractions. Erythrocytes pellets were washed and further lysed using hypotonic phosphate buffer solution (PBS; 1:10 dilution, 0.001 M, pH 7.4) within 10 min at room temperature, after which the samples were again centrifuged on similar parameters. IND concentration in both fractions was measured using HPLC‐DAD technique based on reverse‐phase C18 column with water/acetonitrile elution (0.1% formic acid) and monitoring at λmax. Bioavailability was assessed as % of IND distribution in extracellular and intracellular fractions.

### 2.9 Measurement of Key Insulin Signaling Pathway Activity as AMPK

The activity of AMPK in the cell‐free system was assessed by the spectrophotometric coupled‐enzyme assay. Human AMPK enzyme (SRP5003, Sigma, 0.05 µg/µL) was dissolved in the buffer that consisted of 50 mM HEPES, 10 mM MgCl2, 0.2 mM AMP, 0.5 mM ATP, 1 mM phos‐phoenolpyruvate, 0.15 mM NADH, 0.2 mg/mL pyruvate kinase, and 0.2 mg/mL lactate dehydrogenase. The AMPK enzyme was incubated at 37°C for 5 min to reach the equilibrium with the reaction medium. Next, the AMPK reaction was started by the addition of the AMPK‐specific peptide substrate SAMS peptide (Sigma, S4878, 1 mM), and the oxidation of NADH was registered as the reduction in its absorption at 340 nm. AMPK activity was calculated as nmol NADH oxidized per minute per mg protein (Hawley et al. 1996).

### 2.10 AKT Activity Determination

The activating effect of the compound IND and reference compound SC79 was established according to the procedure of Alessi et al. (1996). This procedure involves the incubation of the human Akt enzyme in vitro (Sigma‐Aldrich, C8482). To create the assay solution, there were used 8 mL of Tris‐HCl buffer (50 mM, pH = 7.5), 1 mL MgCl_2_ (100 mM), 100 µL of ATP (100 mM, A2383, ≥ 99%), 200 µL of phosphoenolpyruvate (PEP, 100 mM), 200 µL NADH (10 mM, Sigma N8129, ≥ 97%), 100 µL Kemptide (10 mM, Sigma C9781; Leu‐Arg‐Arg‐Ala‐Ser‐Leu‐Gly), 2 U of pyruvate kinase, and 3 U of lactate dehydrogenase. The reaction mixture was incubated at 37°C for 20 min, measuring the change in the absorb.

### 2.11 Molecular Docking Analysis

Molecular docking studies were carried out using AutoDock 4.2 to evaluate the binding interactions between IND and the selected target proteins. The three‐dimensional crystal structures of the proteins were retrieved from the Protein Data Bank (PDB). Prior to docking, all water molecules and native ligands were removed, and polar hydrogen atoms as well as Kollman charges were added to the protein structures. The chemical structure of IND was optimized by energy minimization before docking simulations. Grid box parameters were adjusted to cover the active binding sites of the target proteins using AutoGrid tools. Docking simulations were performed using the Lamarckian Genetic Algorithm (LGA) implemented in AutoDock 4.2 with default parameters. Several independent docking runs were conducted to identify the most stable ligand–protein conformations. The binding affinity of IND toward the proteins was evaluated according to the lowest binding energy values (kcal/mol), hydrogen bond interactions, hydrophobic interactions, and ligand orientation within the catalytic site. The best docking conformations were finally visualized using Discovery Studio Visualizer and PyMOL.

### 2.12 Determination of IND and Spermatozoids Glucose Consumption and AMPK and AKT Activities

The spermatozoa sample was obtained from a healthy 25‐year‐old male volunteer after informed consent approved by the National Ethics Committee (TNAPO‐06‐B‐071; approval number TNC#2025‐3125). The sample was isolated by density gradient centrifugation (Percoll 40%–80%, 400 × g, 20 min) and washed by centrifugation (400 × g, 10 min). Cells were then resuspended in HTF (human tubal fluid) medium to a final concentration of 2 × 10^7^ spermatozoa/mL. Preincubation was performed in HTF medium containing IND (0.5–50 µg/mL) for 4 h at 37°C under gentle agitation. Untreated HTF medium served as the negative control, while IND‐treated samples were used as experimental conditions. Samples were collected every 2 h, centrifuged (1500 × g, 10 min), and glucose consumption was quantified using the GOD–POD method (Biolabo, ref. 87409) by measuring absorbance at 505 nm. In parallel, AMPK and AKT activities were evaluated after 24 h of incubation using sperm lysates prepared in ice‐cold lysis buffer (Tris‐HCl, NaCl, Triton X‐100, and protease/phosphatase inhibitors). After centrifugation at 12,000 × g for 10 min at 4°C, supernatants were collected and analyzed for enzyme activity.

### 2.13 Statistical Analysis

The theoretical IND production was estimated from three independent model runs, whereas the experimental production assays were performed in four independent replicates. Experimental data are expressed as the mean ± standard deviation (SD). Statistical significance was determined using one‐way ANOVA followed by the appropriate post hoc test, with *p* < 0.05 considered statistically significant. Statistical analyses were performed using StatView version 5.0 (SAS Institute Inc., Cary, NC, USA).

## 3 Results

### 3.1 Effect of Culture Medium on IND Production

From the results provided in Table 1, the effect of the culture medium on both BL21‐DE3 biomass growth and IND production was found to be significant. Standard LB medium allowed for biomass growth of 1.14 g/L/24 h, followed by NB (0.94 g/L/24 h) and PGB (2.76 g/L/24 h). As far as IND production is concerned, LB medium produced 75.8 mg/L/24 h, compared to a lesser production of 49.8 and 38.9 mg/L/24 h in NB and PGB, respectively.

**Table 1:** Comparison of physicochemical and nutritional culture conditions between LB and LB‐OP and their effect on IND production.

Parameter	LB	LB‐OP
Tryptone (%)	1.00	1.52
Yeast extract (%)	0.50	0.81
Glucose (%)	0.00	1.09
NaCl (%)	1.00	0.875
MgSO_4_·7H_2_O (%)	0.00	0.050
KH_2_PO_4_ (%)	0.00	0.100
pH	7.00	7.80
Temperature (°C)	30	29
Incubation time (h)	24	24
Agitation speed (rpm)	180	200
Biomass (g/L)	2.10	2.30
l‐glutamine (%)	0.00	1.13
Biomass growth (g/L/24 h)	1.04 ± 0.12	2.76 ± 0.31
IND production (mg/L/24 h)	75.8	177.4

### 3.2 Final Optimization of IND Production Using CCD

Following the preoptimization stage, B (glucose), C (tryptone), and D (yeast extract) were recognized as the most influencing factors of IND production. The interaction of these three variables and their effect on IND production is demonstrated in Figure 1. Response surface and contour plot analysis revealed that the optimized values for these three parameters would be 1.02% and 1.52% for glucose and tryptone, respectively, as well as 0.81% for yeast extract (Figure 3D). With this optimal condition, the response surface model predicted that the maximum amount of IND production would be about 180 mg/L/24 h, whereas the experimentally obtained value was 177.4 mg/L/24 h.

**Figure 1:**
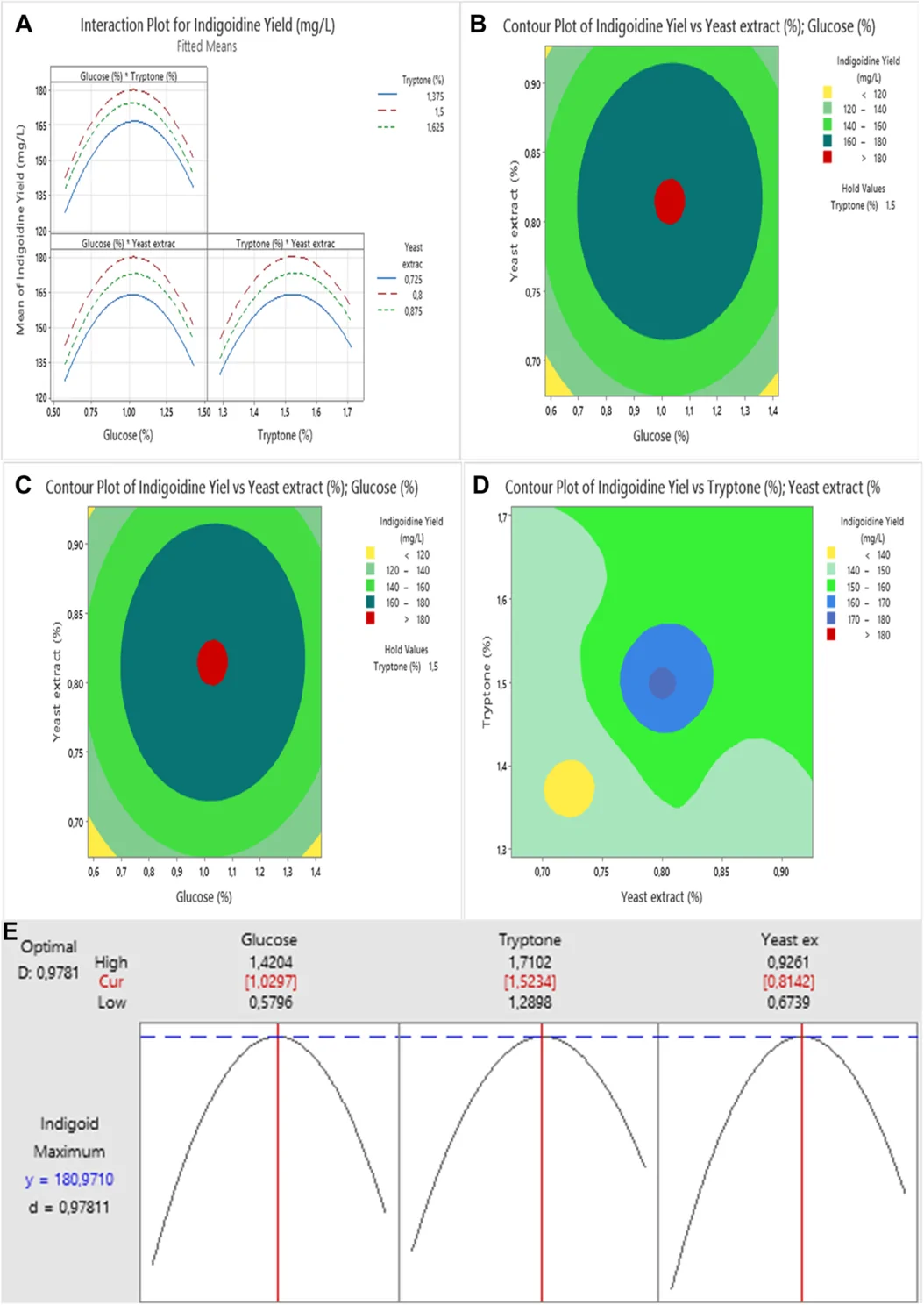
(A–E) Optimization using response surface methodology (RSM) for IND production. It was observed from the interaction and contour graphs that glucose concentration, tryptone concentration, and yeast extract concentration had a profound effect on the production of IND. Based on optimization results, a maximum production of IND (180.97 mg/L/h) was estimated to occur at an optimal value of 0.978.

### 3.3 Comparison Between Standard and Optimized LB Media for IND Production

The changes made in the nutrient concentration and physicochemical conditions of the culture media in LB‐S and LB‐O have led to considerable increases in the IND yield. The optimization was done through an increment of tryptone (1.5%) and yeast extract (0.8%) concentrations, introduction of glucose (1%), and MgSO_4_·7H_2_O and KH_2_PO_4_ in the medium. Also, slight changes in the physicochemical conditions of the culture were made. These included increasing pH to 7.8, lowering temperature to 29°C, and increasing the agitation rate to 200 rpm. These changes helped to increase the biomass growth of BL21‐DE3 from 1.14 to 1.29 g/L/24 h. However, the IND yield rose from 75.8 to 177.4 mg/L/24 h in the optimized medium, which represents a 2.34‐fold increment (Table 1 and Figure 2).

**Figure 2:**
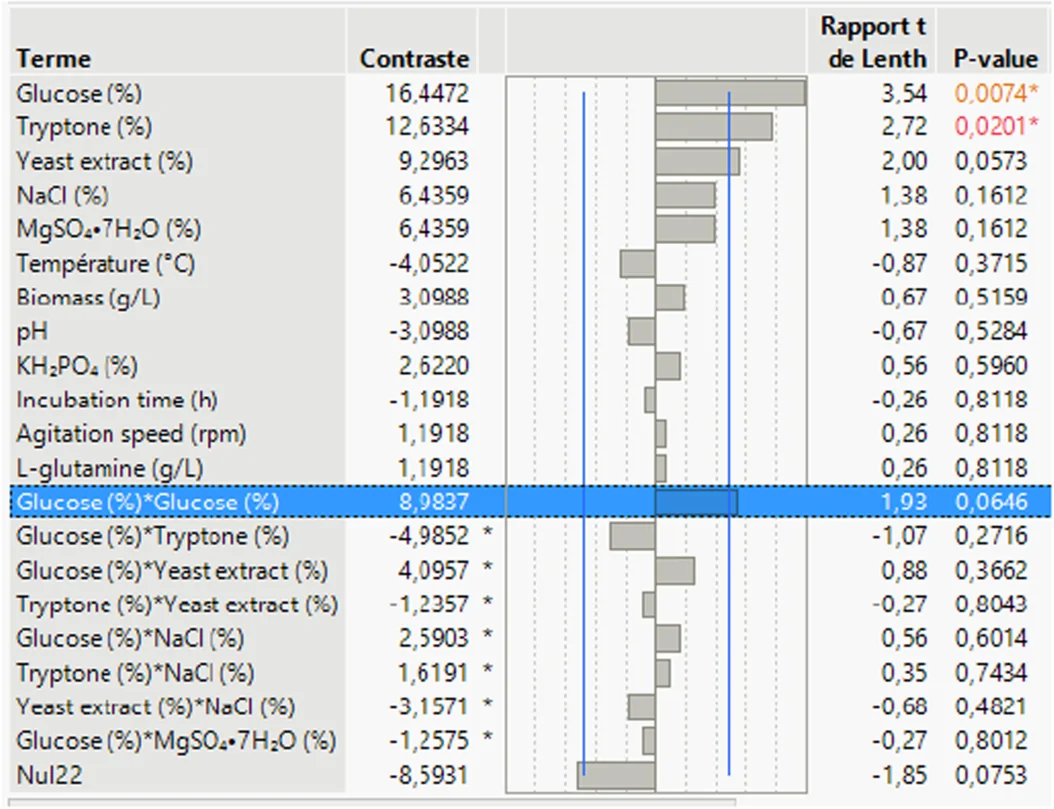
Pareto diagram representing the impact of medium components and growth conditions on the biosynthesis of IND (IND). Glucose and tryptone turned out to be the factors with the greatest influence (*p* < 0.05), while all the remaining factors had no effect. The positive/negative values represent stimulation/retardation effect.

### 3.4 Purification and Identification of IND by HPLC

Figure 3A displays the LC–MS spectrum with HPLC‐DAD spectrum analysis of IND, indicating the occurrence of a significant peak belonging to the target pigment along with the presence of insignificant quantities of the secondary compounds. The resultant spectra indicated the purity level of 92.3% for the pigment. This was additionally verified through UV‐visible spectroscopy analysis (Figure 3B) of IND that displayed the typical absorption spectrum pattern of the pigment with an absorbance maximum at 612 nm.

**Figure 3:**
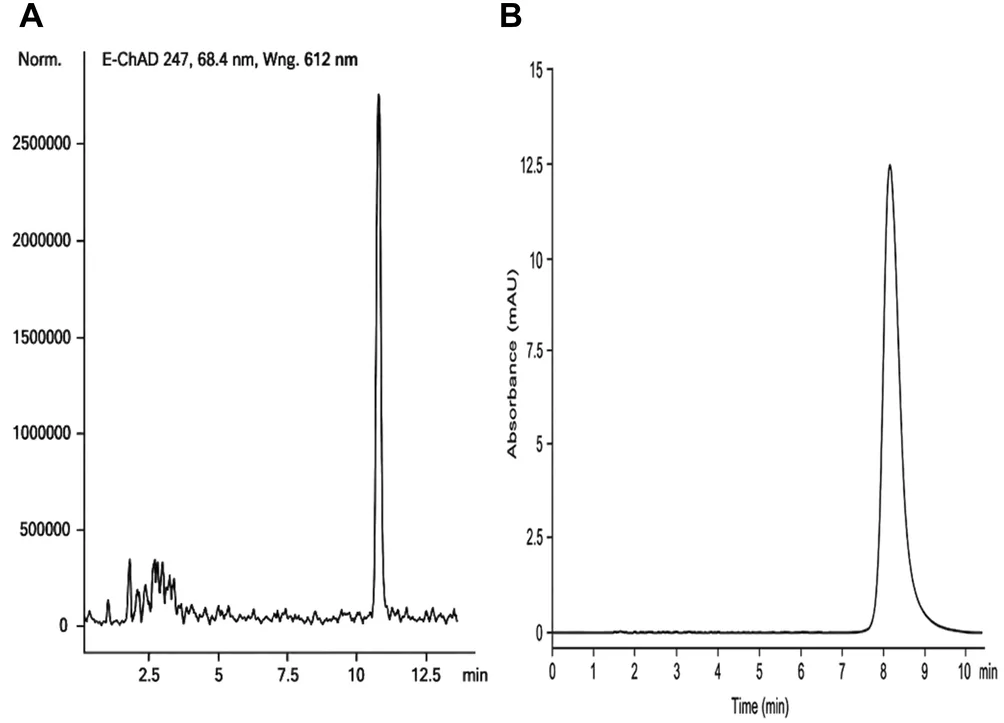
Characterization of purified IND (IND). (A) HPLC chromatogram coupled with LC–MS analysis showing a predominant IND peak, confirming its successful purification and identity. (B) UV–Visible absorbance spectrum of purified IND displaying a characteristic maximum absorbance at 612 nm, further confirming pigment purity and structural integrity.

### 3.5 Evaluation of IND Stability in Relation to Food Technological Indices

The stability of IND was greatly dependent on factors such as pH, temperature, storage, and exposure to light. At pH 7 (neutral), IND displayed moderate stability, where its half‐life was estimated to be about 150 h. At pH extremes, however, IND showed great instability, having t_1_/_2_ values of only 3 and 10 h at pH 1 and 12, respectively. Temperature played an important role in the stability of the drug; low temperatures greatly stabilized it, as the t_1_/_2_ was measured to be 240 h at 4°C. On the other hand, high temperatures caused rapid degradation, with t_1_/_2_ as low as 2 h being attained at 100°C. The stability of the drug decreased over storage time, as indicated by the reduction in t_1_/_2_ from 98 weeks in initial conditions to about 2 weeks over time (after 11–13 weeks). Light was a significant factor in stability, and IND was most stable in darkness, t_1_/_2_ being 220 h, moderately stable under ambient light, where t_1_/_2_ was 100 h, and highly unstable under direct light or UV light, where t_1_/_2_ dropped to 20 h (Table 2).

**Table 2:** Evaluation of IND stability under different food technological conditions (pH, temperature, storage, and light exposure).

pH	1	2	3	4	5	6	6.5	7.8	8	9	10	11	12
pH stability (t½, h)	3	7	12	20	48	110	136	150	120	72	48	24	10
Temperature (°C)	0	2	4	15	24	30	37	50	60	70	80	90	100
Temp stability (t½, h)			240	200	150	120	96	48	30	18	10	5	2
Storage (weeks)	1	2	3	4	5	6	7	8	9	10	11	12	13
Storage stability (t½, w)	98	96	92	86	77	65	44	36	18	9	6	2	2
Light condition	Dark						Amb				UV		
Light stability (t½, h)	220						100				20		

### 3.6 Effect of IND at Different Concentrations on Erythrocytes: Toxicity Assay

From our experiments, we were able to establish that IND showed negligible cytotoxicity towards human erythrocytes within the concentration range examined (0.5–50 µg/mL). Within the lower concentration range of 0.5–10 µg/mL, the percentage hemolysis was below 2%. At intermediate concentrations (15–30 µg/mL), a slight dose‐dependent increase was observed, with hemolysis values ranging from 2.5% to 4.1%, while remaining within acceptable limits. Even at the highest tested concentration (50 µg/mL), hemolysis reached only 5.29%, remaining within the generally accepted nontoxic threshold (< 5%–10%) for erythrocyte‐based assays (Figure 4A).

**Figure 4:**
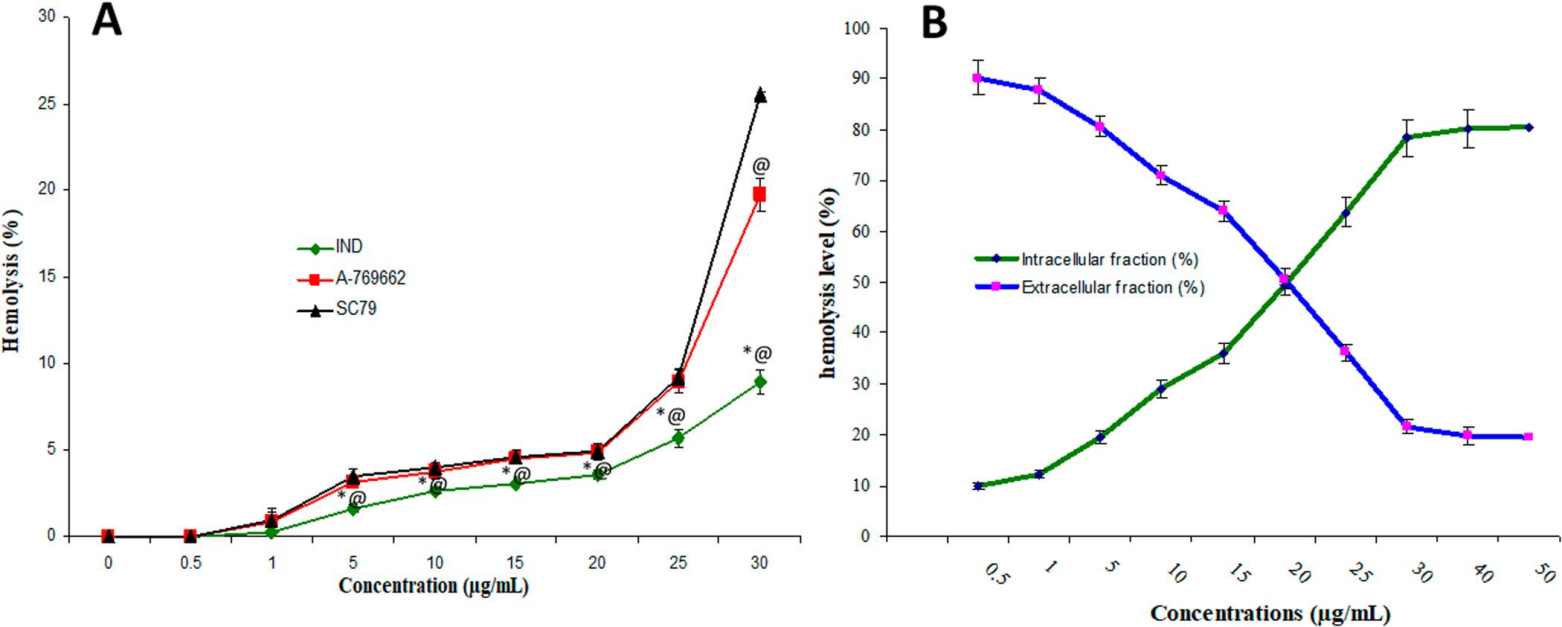
Impact of IND on erythrocyte toxicity and membrane bioavailability. (A) Erythrocyte hemolytic effects of IND treatment in humans at varying concentrations of 0.5–50 µg/mL. (B) Ratio of IND concentrations found outside and inside erythrocyte cells. Statistical analysis as *p < 0.05 versus SC79; ^@^ *p* < 0.05 versus A‐769662.

### 3.7 Evaluation of the Membrane Bioavailability of IND

As shown in Figure 4B, IND exhibits a concentration‐dependent distribution between extracellular and intracellular compartments. At low concentrations (0.5–1 µg/mL), IND is predominantly associated with erythrocytes, with cellular fractions of 90.2% and 87.7%, while the extracellular fraction remained between 9.8% and 12.3%. As the concentration increased (5–15 µg/mL), a progressive decrease in the intracellular fraction was observed, ranging from 80.6% to 64%, accompanied by a corresponding increase in the extracellular fraction. At intermediate concentrations (20–25 µg/mL), the intracellular fraction further decreased to 50.6% and 36.2%, respectively, while the extracellular fraction increased proportionally. At higher concentrations (30–50 µg/mL), IND was mainly detected in the extracellular medium (78.4%–80.5%), while the intracellular fraction stabilized at lower values between 19.5% and 21.6%.

### 3.8 Effect of IND on α‐Amylase Activity

IND, a bacterial pigment, exhibited a significant dose‐dependent inhibitory effect on α‐amylase activity, a key enzyme involved in carbohydrate digestion and closely associated with type 2 diabetes (T2D). Indeed, its inhibitory activity increased progressively with increasing concentration, rising from 12.4% at 0.2 µg/mL to 46.8% at 0.6 µg/mL and reaching a maximum inhibition of 94.6% at 2.6 µg/mL. The IC_50_ value of IND was estimated at 0.69 µg/mL, compared with 0.36 µg/mL for the reference inhibitor Acarbose. This inhibitory effect was further supported by in silico molecular docking analysis, which revealed a binding energy of −7.4 kcal/mol, close to that of Acarbose (−7.7 kcal/mol), indicating a strong affinity toward the active site of α‐amylase. Furthermore, IND established several stabilizing interactions with catalytic site residues, including hydrogen bonds with ASP197, ASP300, and GLN63, as well as π–π interactions with TRP59, demonstrating the stability and strength of the IND–enzyme complex (Figure 5 and Table 3).

**Figure 5:**
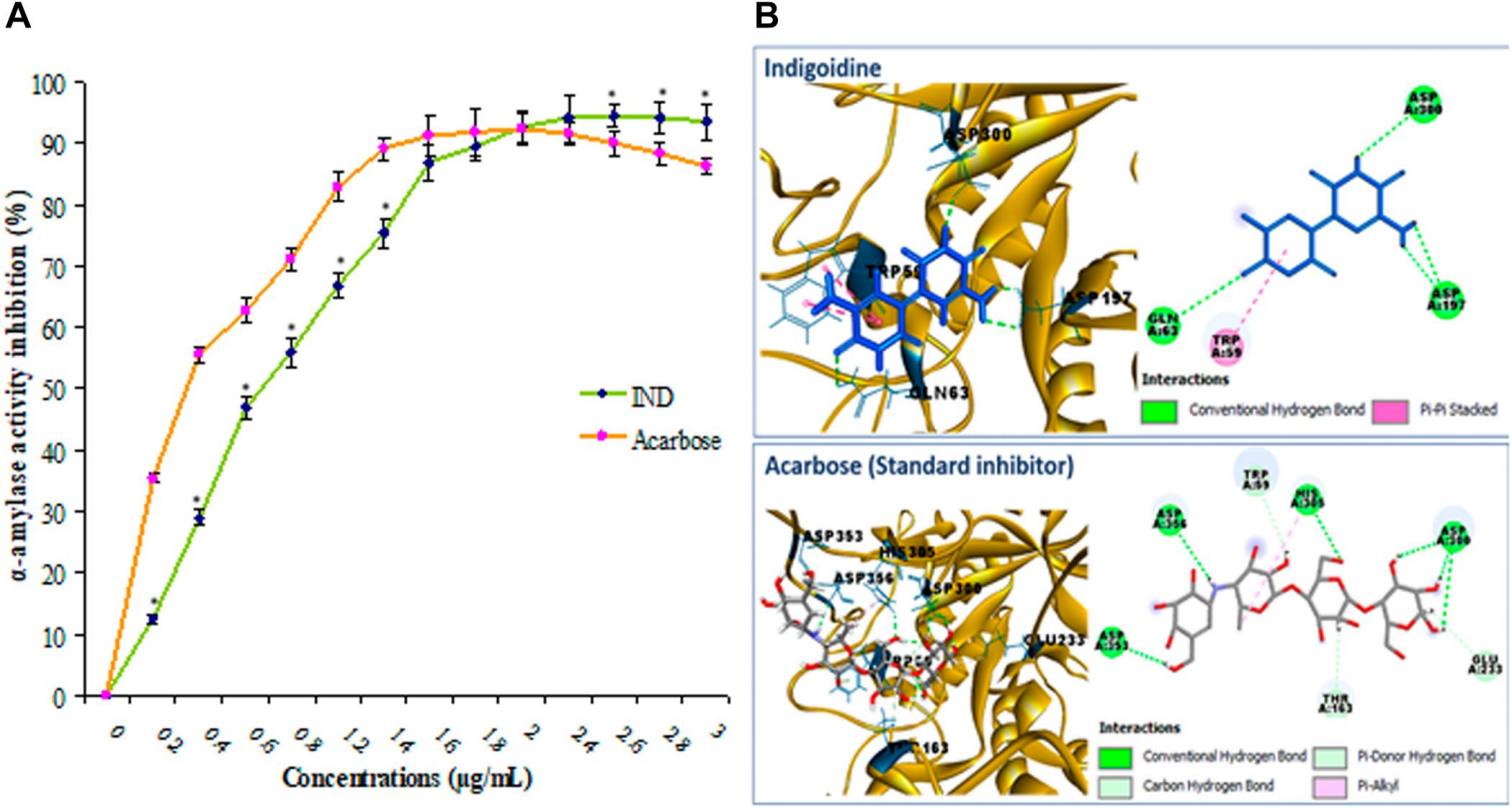
Effect of IND on α‐amylase activity and molecular docking analysis. (A) IND dose‐dependently inhibited α‐amylase activity, with an IC_50_ of 0.69 µg/mL compared with 0.36 µg/mL for acarbose. (B) Docking analysis revealed strong binding of IND to the α‐amylase active site (−7.4 kcal/mol), involving key interactions with ASP197, ASP300, GLN63, and TRP59. * Indicates a statistically significant difference between IND and the standard inhibitor (*p* < 0.05).

**Table 3:** Comparison of in vitro biological activities and molecular docking binding energies of IND and reference compounds against metabolic target enzymes.

Target enzyme	Compound	Biological activity	IC_50_/EC_50_ (µg/mL)	Binding energy (kcal/mol)
α‐amylase	IND	Inhibition	0.69 ± 0.028*	−7.4
	Acarbose	Inhibition	0.36 ± 0.016	−7.7
lipase	IND	Inhibition	0.37 ± 0.026*	−8.1
	Orlistat	Inhibition	0.62 ± 0.014	−6.9
AMPK	IND	Activation	1.36 ± 0.06*	−6.9
	Ligand 992	Activation	0.98 ± 0.04	−9.8
AKT	IND	Activation	4.59 ± 0.18*	−5.5
	SC79	Activation	3.20 ± 0.14	−5.6

* Indicates a statistically significant difference between IND and the standard inhibitor (*p* < 0.05).

### 3.9 Effect of IND on Lipase Activity

For the first time, our study demonstrates that IND, a bacterial pigment, exerts a significant and dose‐dependent inhibitory effect on pancreatic lipase activity, a key enzyme involved in lipid digestion and closely associated with obesity and metabolic disorders. This inhibitory effect increased progressively with increasing IND concentration, reaching a maximum inhibition of 93.6% at 2.6 µg/mL. The IC_50_ value of IND was determined to be 0.37 µg/mL, indicating a strong inhibitory activity compared with the reference inhibitor Orlistat (ORL), which exhibited an IC_50_ value of 0.62 µg/mL. This potent anti‐lipase activity of IND was further confirmed by molecular docking analysis, which revealed a strong binding affinity toward pancreatic lipase, characterized by a binding energy of −8.1 kcal/mol, exceeding that of ORL (−6.9 kcal/mol). Furthermore, the in silico study showed that IND strongly interacts with the enzyme active site through several hydrogen bonds involving ASP79, ARG256, and GLY76, as well as hydrophobic and π‐type interactions with PHE77, HIS263, ALA260, and LEU264 residues (Figure 6 and Table 3).

**Figure 6:**
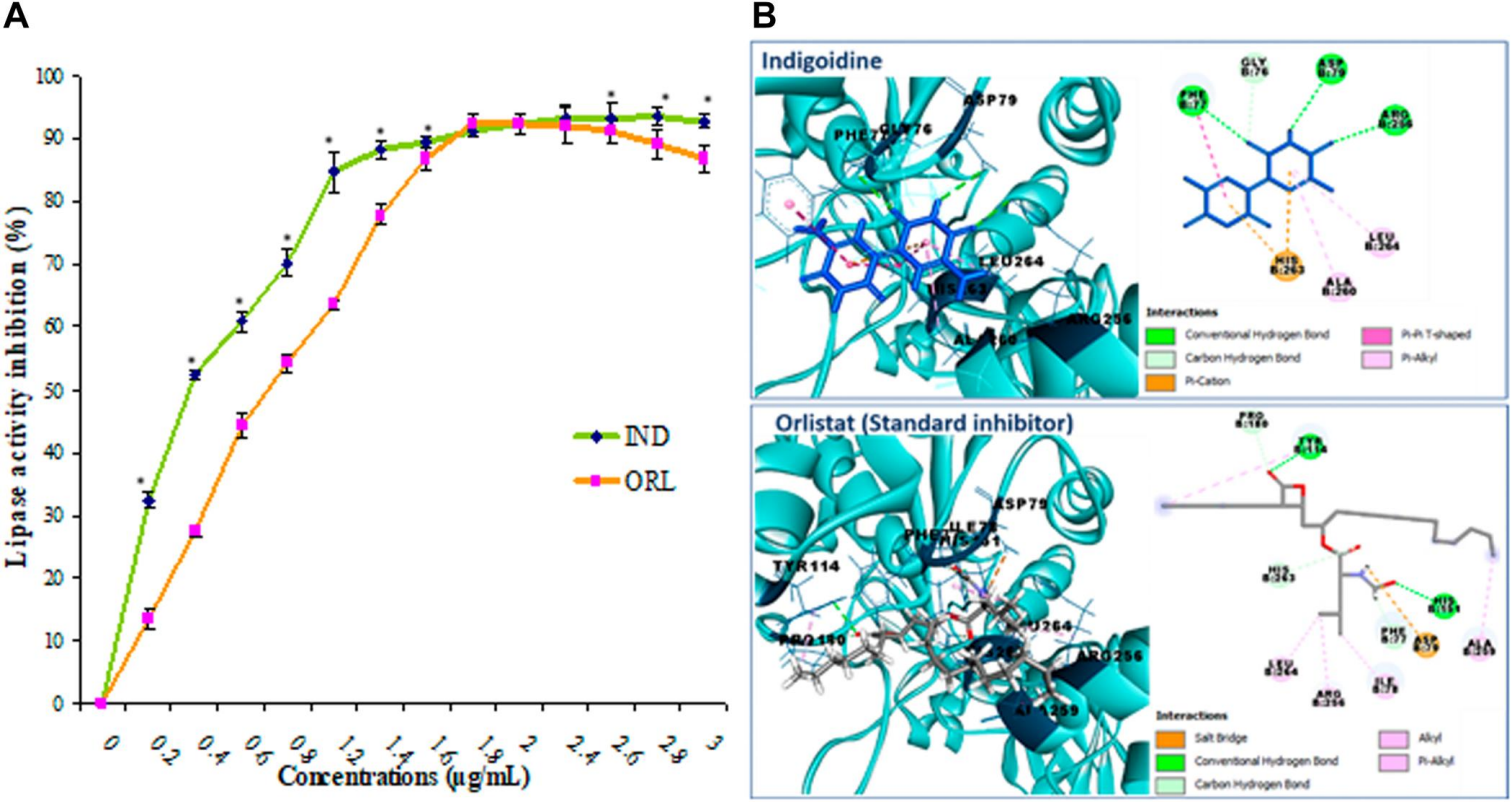
Action of IND on pancreatic lipase and docking studies. (A) Pancreatic lipase activity was concentration‐dependently inhibited by IND, and the inhibition had an IC_50_ value of 0.36 µg/mL, while the IC_50_ value for orlistat was 0.62 µg/mL. (B) The docking studies showed strong interaction between IND and the active site of lipase (−8.1 kcal/mol) through hydrogen bonding with ASP79, ARG256, and GLY76, and hydrophobic interaction with PHE77, HIS263, ALA260, and LEU264. * Indicates a statistically significant difference between IND and the standard inhibitor (*p* < 0.05).

### 3.10 Effect of IND on the Activity of Key Enzyme Related to Starch Catabolism as AMPK

As shown in Figure 8, IND exhibited a dose‐dependent stimulatory effect on AMPK activity, with activation increasing from 1.94% at 0.1 µg/mL to 65.1% at 2.8 µg/mL, followed by an enzymatic saturation phase characterized by a slight and gradual decline in activation (Figure 7A). This stimulatory effect was further supported by an EC_50_ value of 1.36 µg/mL, compared with 0.98 µg/mL for the reference activator A‐769662. The AMPK‐activating potential of IND was also confirmed by an in silico molecular docking study, which revealed that IND interacts with the active site of AMPK with a binding energy of −6.9 kcal/mol, compared with −9.8 kcal/mol for the specific activator A‐769662. Molecular interaction analysis showed that IND binds to the AMPK activation site through several types of interactions, including conventional hydrogen bonds with residues ARG B107 and LEU A18, carbon–hydrogen bond interactions involving SER B103 and ARG B83, a π‐cation interaction with LYS A31, and two π‐alkyl interactions with VAL B113 and VAL A11 (Figure 7B and Table 3).

**Figure 7:**
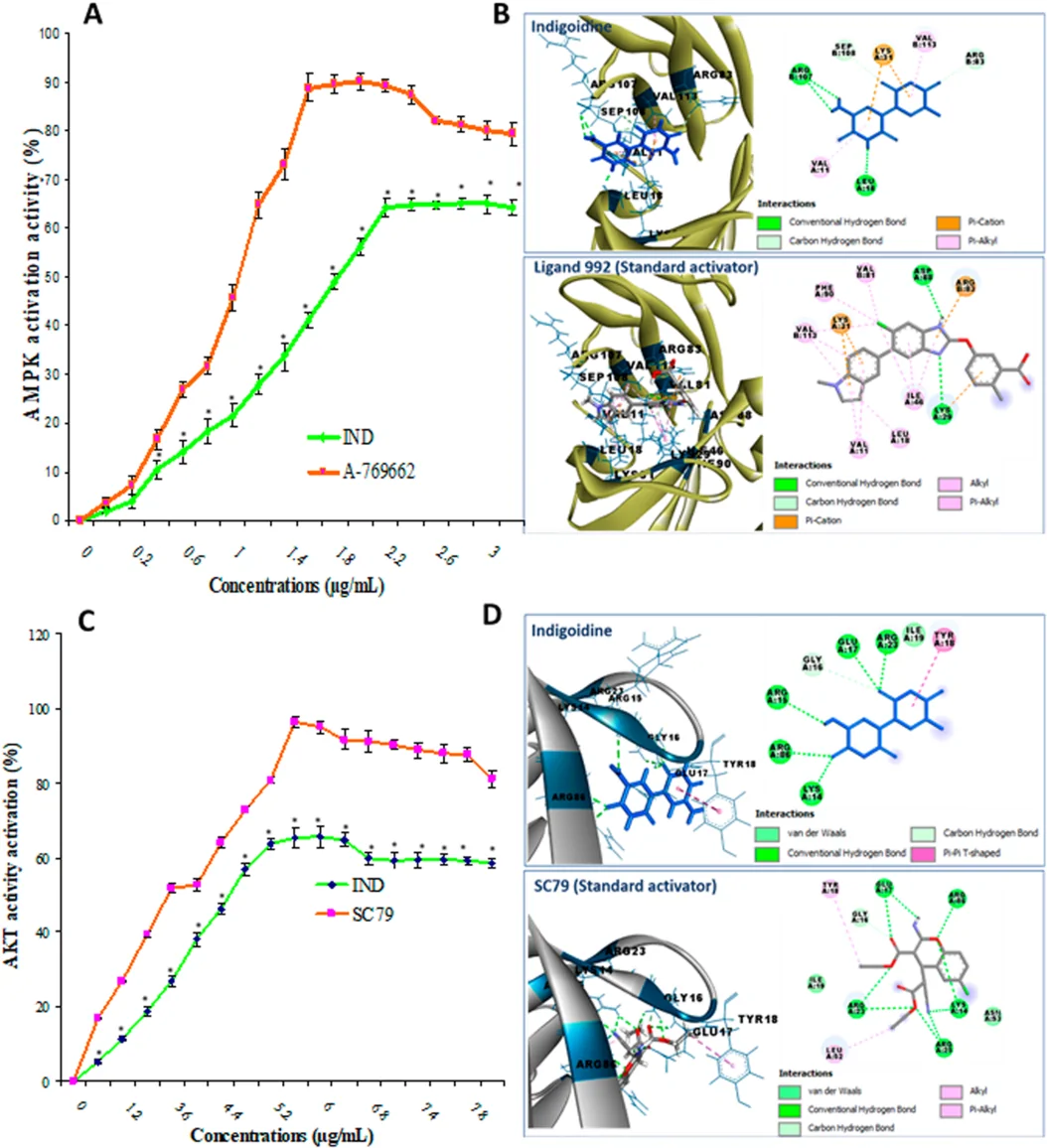
Influence of IND on the activities of AMPK and AKT and molecular docking studies. (A, B) IND demonstrated a concentration‐dependent activation of AMPK with an EC_50_ value of 1.36 µg/mL, as well as good binding to the AMPK activation site (−6.9 kcal/mol). (C, D) Similarly, IND induced AKT activity with an EC_50_ value of 4.59 µg/mL, and possessed excellent affinity with AKT active site (−5.5 kcal/mol), similar to that of standard activator SC79. * Indicates a statistically significant difference between IND and the standard inhibitor (*p* < 0.05).

### 3.11 Effect of IND on AKT Activity, a Key Enzyme in Insulin Signaling

As shown in Figure 7C, IND stimulated AKT activity in a dose‐dependent manner. The activation increased from 5.1% at 0.5 µg/mL to 65.6% at 6 µg/mL. Beyond this concentration, a slight decline in activity was observed, suggesting the onset of enzymatic saturation. This activating effect was further supported by an EC_50_ value of 4.59 µg/mL, compared with approximately 3.2 µg/mL for the specific AKT activator SC79. Furthermore, molecular docking analysis revealed that IND interacts with the active site of AKT with a binding energy of −5.5 kcal/mol, which is very close to that observed for SC79 (−5.6 kcal/mol). The interaction profile showed the formation of several hydrogen bonds with residues ARG23, GLU17, ARG15, ARG86, and LYS14, as well as carbon–hydrogen interactions involving GLY16, π–π interactions with TYR18, and a van der Waals interaction with ILE19 (Figure 7D and Table 3).

### 3.12 Effect of IND on Spermatozoids Glucose Consumption

As shown in Figure 8A, our results demonstrate that IND induces a dose‐dependent stimulation of glucose utilization in human spermatozoa. Compared with the control (100%), glucose consumption progressively increased from 103.2% at 0.5 μg/mL to 106.1% at 1 μg/mL, reaching 111.8%, 118.9%, 126.8%, and 133.4% at 5, 10, 15, and 20 μg/mL, respectively. A maximal stimulation of 128.7% was observed at 25 μg/mL. Apart from this concentration, glucose consumption showed gradual decrease with percentages of 123.9%, 98.9%, 95.1%, and 90.1% at concentrations of 30, 35, 40, and 50 μg/mL, respectively, suggesting the commencement of cytotoxicity based on reduced metabolism activity. On the other hand, A‐769662 caused an initial rise in glucose consumption to a maximum value of 135.8% at concentration of 20 μg/mL from 112.3% at 0.5 μg/mL. Nevertheless, activation of glucose consumption was later reversed by increasing concentrations, with the consumption falling from 106.8% at 25 μg/mL to a minimum of 16.5% at 50 μg/mL. Similarly, SC79 caused increases in glucose consumption at low and medium concentrations to reach maximum of 131.8% at 15 μg/mL, while higher concentrations of SC79 had a significant inhibiting effect on glucose consumption as values fell to 85.4%, 42.8%, 22.3%, 14.7%, and 8.4% at concentrations of 25, 30, 35, 40, and 50 μg/mL, respectively. From these results, it can be deduced that IND activates glucose metabolism significantly and with low toxicity compared to A‐769662 and SC79 at high concentrations.

**Figure 8:**
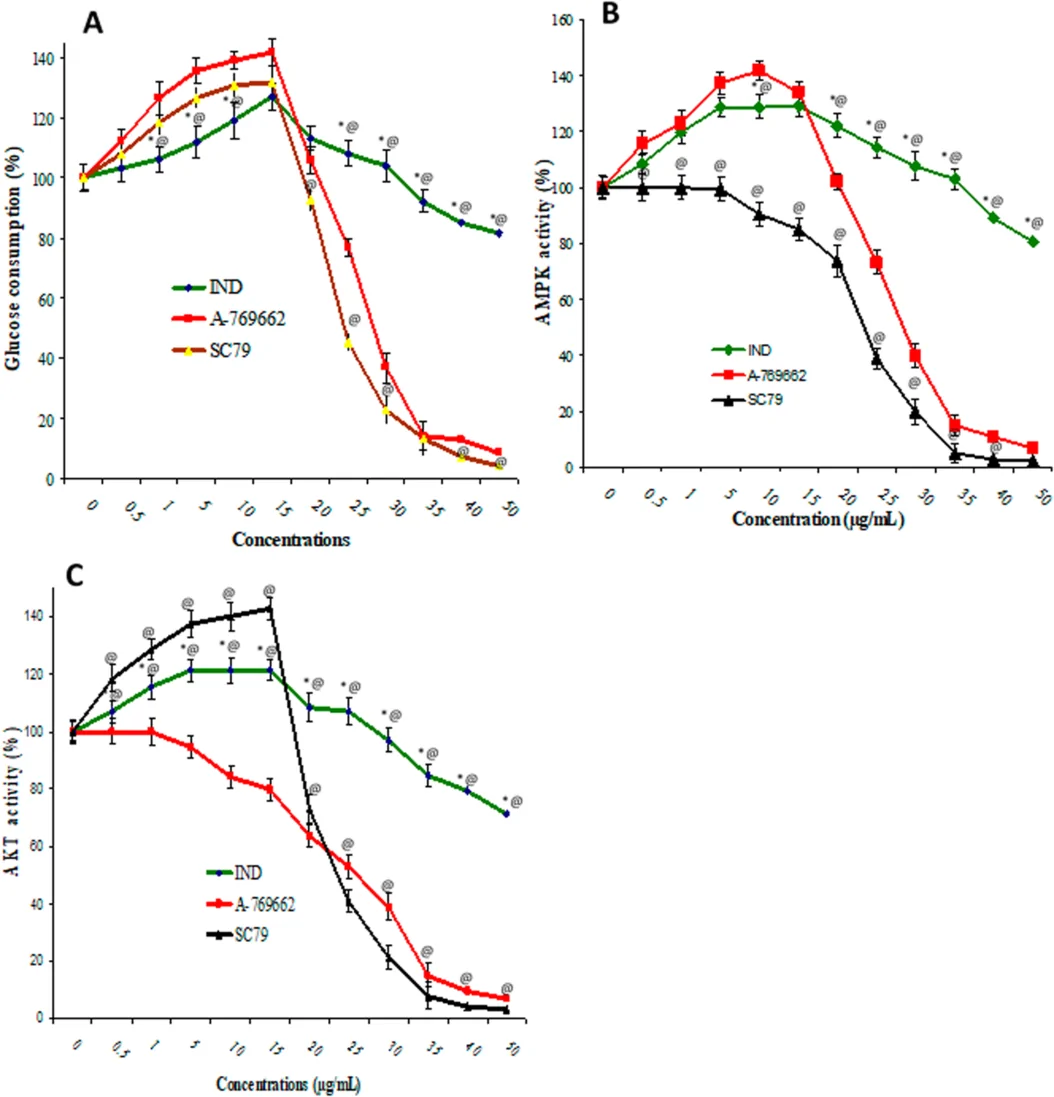
Effect of IND on glucose uptake and signaling pathways involved in glucose metabolism in human spermatozoa. (A) The concentration of IND was found to significantly increase glucose uptake in a dose‐dependent manner, showing maximum effects at 20–25 µg/mL with lesser toxicity compared to the two activator drugs, A‐769662 and SC79. (B) Maximum activation of AMPK was seen by the drug at a concentration of 15 µg/mL. (C) The activation of AKT shows that the insulin‐signaling pathway is also activated by the drug. Statistical analysis as *p < 0.05 versus SC79; ^@^ *p* < 0.05 versus A‐769662.

### 3.13 Effect of IND on Spermatozoids AMPK Activity

As presented in Figure 8B, IND activates AMPK signaling in human spermatozoa in a dose‐dependent manner. The AMPK activity progressively increased from 108.5% at 0.5 µg/mL to a maximum of 128.9% at 15 µg/mL relative to the control (100%). This result indicates the effective stimulation of AMPK signaling pathway by IND treatment. Further increase of the IND dose to 20–35 µg/mL leads to a decrease in AMPK activity, which can be attributed to its toxicity resulting from partial loss of cellular vitality and motility of sperm cells. In turn, A‐769662 demonstrated even more potent AMPK activation up to a peak of 141.7% at 15 µg/mL dose. However, the further increase of the dose results in a strong decrease in AMPK activity to 72.9%, 39.7%, and 14.8% at 25, 30, and 35 µg/mL, respectively. In contrast, SC79 did not induce AMPK activation at lower concentrations, with activity values remaining close to those of the control. As the concentration increased, a progressive decline in AMPK activity was observed, reaching 84.2% at 10 µg/mL, 38.7% at 25 µg/mL, and 4.9% at 35 µg/mL. These findings suggest that IND effectively activates AMPK in human spermatozoa while exhibiting lower toxicity than A‐769662 at high concentrations, whereas SC79 does not appear to exert any stimulatory effect on this metabolic signaling pathway.

### 3.14 Effect of IND on Spermatozoids AKT Activity

As shown in Figure 8C, IND induced a dose‐dependent activation of AKT activity in human spermatozoa. Compared with untreated control spermatozoa (100%), AKT activity progressively increased from 106.8% at 0.5 µg/mL to a maximum of 121.4% at 15 µg/mL, indicating a moderate stimulation of the AKT signaling pathway. At higher concentrations (20–35 µg/mL), a slight decline in activity was observed, reaching 108.4% and 84.7%, respectively, suggesting the onset of a saturation effect or a partial reduction in cell viability at elevated doses. In comparison, A‐769662 did not stimulate AKT activity at low concentrations and instead induced a progressive dose‐dependent inhibition, with activity decreasing to 14.9% at 35 µg/mL. In contrast, SC79 produced a strong activation of AKT at low and intermediate concentrations, with activity increasing from 118.4% at 0.5 µg/mL to a maximum of 142.8% at 15 µg/mL. In addition to the mentioned level of activity, SC79 displayed notable levels of cytotoxicity, which is confirmed by considerable inhibition of AKT activity, amounting to 72.9% at the level of 25 µg/mL and reaching even 7.8% when administered in the concentration of 35 µg/mL. According to the results presented above, it may be concluded that IND demonstrates the ability to activate AKT‐dependent metabolic pathways with a considerably higher activity and stability compared to SC79. Additionally, unlike A‐769662, the compound under analysis can exhibit activating properties at higher concentrations.

## 4 Discussion

In the current study, an attempt was made to design an optimal medium to enhance bacterial growth and the maximum production of IND by nonpathogenic recombinant bacteria of *E. coli* BL21‐DE3, which can be used in the future in food and pharmaceutical industries. At first, the bacterium was cultivated in three different media for 24 h. It was found out that the maximum bacterial growth was achieved in PGB medium (2.76 g/L/24 h), while the maximum IND production was achieved in LB medium (75.8 mg/L/24 h). These high levels of productivity might be explained by the highly nutritive properties of LB medium, containing the necessary sources of carbon and nitrogen needed for bacterial growth and synthesis of IND. More precisely, tryptone and yeast extract supply amino acids and peptides, which might act as precursors of IND biosynthesis. The results obtained are in agreement with the literature data (Zhao et al. 2026) concerning the potential of LB medium for the efficient IND production. Thus, Zhao et al. (2026) observed increased production of IND in recombinant *E. coli* grown in LB medium, whereas Li et al. (2026) demonstrated its efficiency for IND accumulation.

As it can be observed from our experiment, there really was a rise in the amount of IND formation through the manipulation of the growth conditions. Comparing our experimental results with the results of the LB medium, we have noticed that there is a 2.34‐fold increase in the amount of IND produced (75.8–177.4 mg/L/24 h) with a small rise in the amount of biomass (1.14–1.29 g/L/24 h). First, elevated concentrations of tryptone (1.52%) and yeast extract (0.81%) could promote the synthesis of amino acids, peptides, vitamins, and other growth‐promoting compounds necessary for active metabolism. Even more importantly, addition of l‐glutamine (1.13%), an immediate precursor of IND synthesis, could increase precursor synthesis and contribute to the synthesis of the enzyme BpsA, responsible for pigment production. These results are consistent with the data provided by Chen et al. (2025), who found that soybean okara hydrolysates increased IND synthesis because of the better availability of ATP and CoA, two important cofactors for pigment synthesis.

This could also have been because of the introduction of glucose, MgSO_4_.7H_2_O, and KH_2_PO_4_ into the system. Glucose was a cheap source of carbon and energy that facilitated the production of ATP and precursors. Magnesium ions were introduced by MgSO_4_.7H_2_O, which was necessary for the enzymes’ activities. Finally, phosphates introduced by KH_2_PO_4_ were needed for ATP production and metabolism within the cells (Panchanawaporn et al. 2022; Wehrs et al. 2018; Xu et al. 2015; Zhao et al. 2026). In addition to these studies, it is shown that a balanced supply of carbon along with minerals can positively influence pigment formation by improving the metabolic performance and precursor generation. Similar to the findings of Chen et al. (2025), energy metabolism and cofactor availability seem to influence the formation of IND. It can be speculated that the combination of glucose, MgSO_4_·7H_2_O, and KH_2_PO_4_ may create a conducive environment for IND formation.

Moreover, the optimization of culturing conditions also played an important role in the increase in the rate of pigment formation. The elevation of stirring rate to 200 rpm could have been beneficial in improving oxygen supply and consequently increased respiration and energy supply for pigment synthesis in the form of ATP. As pigment production is dependent on respiration processes, better air supply could have positively influenced pigment accumulation. Cultivation at 29°C is close to the optimal temperature for growth and enzymatic reactions in *E. coli* which is confirmed by recent study of Wehrs et al (2018). Apart from metabolic pathway modification, cultivation conditions also play a significant role in ensuring efficient IND synthesis. Various factors, including aeration, availability of oxygen, temperature, pH, and physiological state of the cell greatly impact the process of synthesis. It was previously reported that effective IND synthesis in recombinant *E. coli*, especially when IDGS is involved, depends greatly on fine‐tuning of the above‐mentioned physicochemical parameters (Banerjee et al. 2020; Zhang et al. 2022). Another important factor was the oxygen level. Molecular oxygen is needed to reoxidize flavin mononucleotide (FMNH_2_) during IND synthesis in the oxidase domain; therefore, low oxygen levels can significantly reduce the production of the pigment Alessi et al. 1996. In the present study, increasing agitation would help increase oxygen transfer and metabolism, resulting in better IND production. Alkaline pH is reported to be favorable for IND biosynthesis as well (Hawley et al. 1996).

Stability is another important attribute that governs the practical use of natural pigments in various industries, including the food and pharmaceutical industry. Stability of IND was found to be very good under milder conditions, maximum being recorded at near neutral pH (pH 7.8; t½ = 150 h); however, low pH (pH 1; t½ = 3 h) and higher alkalinity (pH 12; t½ = 10 h) significantly lowered stability of the pigment, which is in agreement with previous reports on stability of natural pigments reported by (Paik et al. 2001). Temperature also played an important role in the stability of IND, which was highly resistant at temperatures like 4°C (t½ = 240 h) and 30°C (t½ = 120 h), but degraded very quickly at temperatures greater than 60°C. Furthermore, it showed good stability when kept in the dark, whereas it was highly susceptible to light, especially UV light (t½ = 20 h) (Oka et al. 2026).

The bioavailability assay showed that IND possesses excellent capacity for interaction and penetration of human cell membranes. In fact, after treatment with human erythrocytes, a considerable amount of the dye has been found inside the cells, meaning efficient penetration and uptake. This property seems to be associated with the physicochemical characteristics of IND, enabling penetration and accumulation inside the cells. The intracellular availability implies that IND can access intracellular targets and consequently increase its biological activity, thus allowing its application in foods and drugs. Although IND has attracted increasing interest as a natural bacterial pigment, relatively few studies have investigated its production optimization, bioavailability, toxicity, and therapeutic potential. Nevertheless, the available evidence suggests that IND possesses several characteristics that make it a promising candidate for industrial, food, and biomedical applications. According to Cude et al. (2012), beyond its traditional use as an industrial dye, IND can be considered a safe natural colorant for food applications due to its antioxidant and antimicrobial properties. Furthermore, Qi et al. (2026) reported that IND exhibits remarkable physicochemical stability under various environmental conditions, including exposure to light, heat, and pH fluctuations.

Besides being a natural dye, there is a possibility that IND might emerge as a potent bioactive molecule, which can have several applications in the fields of nutraceuticals and pharmaceuticals. Metabolic diseases, comprising obesity, diabetes, and insulin resistance, happen to be the most common chronic noncommunicable illnesses globally and have strong associations with oxidative stress, chronic inflammation, and cardiac ailments. Hence, IND might prove to be an effective choice for exploring its possible antidiabetic and anti‐obesity benefits owing to the rising interest in developing natural therapies instead of artificial drugs. In this regard, future research needs to focus on the possible influence of IND on the key metabolic targets like α‐amylase and pancreatic lipase, apart from its impact on important signaling pathways like AMPK and AKT.

This is the first study to examine the impact of IND on molecular targets involved in the pathophysiology of diabetes, obesity, and insulin resistance. The findings revealed that IND exhibits multitarget biological activity via inhibition of α‐amylase and pancreatic lipase along with activation of AMPK and AKT, important molecules involved in regulation of glucose metabolism and energy production. As regards the inhibition of α‐amylase enzyme, IND shows a high inhibitory effect with IC_50_ of 0.69 µg/mL that is only slightly more than that of acarbose as a reference compound (IC_50_ = 0.36 µg/mL). It can be assumed that IND is a strong inhibitor of carbohydrate hydrolysis and could effectively regulate postprandial hyperglycemia. Besides, the molecular docking studies revealed a binding energy of −7.4 kcal/mol for IND, which is nearly equal to the energy value of −7.7 kcal/mol for acarbose as the reference, suggesting that IND is capable of forming stable complexes with α‐amylase due to hydrogen bonding with ASP197, ASP300, and GLN63 as well as π–π interactions with TRP59. None of the studies have explored the possible inhibition of IND on α‐amylase, which is an important enzyme responsible for digestion and hyperglycemia after eating. The antidiabetic properties of other pigments have been described; one such pigment is prodigiosin, which inhibits both α‐amylase and pancreatic lipase in vitro and silico showed by the study of El‐Sofany et al. (2025). In addition, it was shown to have an insulin‐regulating effect due to the inhibition of PTP1B and DPP‐4 enzymes. All these results were validated using diabetic rats in vivo, where prodigiosin decreased pancreas inflammation and oxidative stress and β‐cell count (El‐Sofany et al. 2025b).

On the other hand, the compound IND proved to be a better inhibitor against lipase compared to ORL, which is the reference drug used for comparison. For instance, IND possessed an IC_50_ value of 0.37 µg/mL compared to 0.62 µg/mL for ORL, suggesting that it was more effective in blocking pancreatic lipase. The claim was further supported by the results obtained from molecular docking, which revealed that IND had a greater binding affinity (−8.1 kcal/mol) compared to ORL (−6.9 kcal/mol). Moreover, IND formed many stabilizing interactions with various key amino acid residues in the active site, such as ASP79, ARG256, GLY76, PHE77, HIS263, ALA260, and LEU264. No previous study showed the effect of IND on lipases, as far as we know. However, bacterial pigments perfectly interacts with pancreatic lipase and prevents and protects against against obesity, hyperlipidemia, cardiovascular diseases, and inflammation as Monascus fungus pigments (Gao et al. 2024), prodigiosin (El‐Sofany et al. 2025), and lipstatin from *Streptomyces toxytricini* (Dubey 2022).

Besides inhibitory effects on α‐amylase and pancreatic lipase, IND showed remarkable stimulatory effects on the activity of two vital enzymes, AMPK and AKT. There was a dose‐dependent increase in the AMPK activity of 65.1% at a concentration of 2.8 µg/mL with EC_50_ equal to 1.36 µg/mL, which was relatively close to the EC_50_ value of the reference AMPK activator, A‐769662 (EC_50_ = 0.98 µg/mL). Despite the docking score value of IND being smaller than A‐769662 (−6.9 vs. −9.8 kcal/mol), this pigment had many interacting bonds with critical amino acid residues of the activation site in AMPK enzyme, namely, hydrogen, π‐cation, and π‐alkyl bonds. In fact, our study is the first investigation to evaluate and demonstrate this activating effect of AMPK, regulator of cellular energy homeostasis and a major therapeutic target for obesity, diabetes, and insulin resistance by IND. Previous studies have established that bacteria‐produced dyes significantly stimulate AMPK activity and facilitate insulin sensitivity and fight against diabetes similar to how the Monascus dyes enhance lipid metabolism, inhibit adipogenesis, and mitigate hyperlipidemia owing to the activation of AMPK, as elucidated by Hsu et al. (2014); prodigiosin has proven useful in improving glucose and lipid metabolism and might exert an impact on the key metabolic pathways, as proposed by El‐Sofany et al. (2025); Monascus pilosus SWM‐008 red mold rice along with its constituents – monascinol and monascin ‐ inhibit obesity in the high fat‐fed rats due to the synergistic influence of gut microbiota and anti‐lipogenesis effect, as shown by Liu et al. (2025).

Likewise, IND activated AKT in a dose‐dependent manner and reached maximum activity (65.6%) at 6 µg/mL concentration. Despite having a slightly higher EC_50_ value (4.59 µg/mL) compared to the specific activator SC79 (around 3.20 µg/mL), molecular docking analysis showed similar binding affinity (−5.5 and −5.6 kcal/mol, respectively). Additionally, IND established various interaction links with critical amino acid residues within the active site of AKT, such as ARG23, GLU17, ARG15, ARG86, and LYS14. As the binding affinity of IND and SC79 were nearly equal, this implies that the activation of AKT by IND is likely through a mechanism analogous to that of SC79. This is because AKT is important for insulin signaling, glucose transportation, glycogen formation, and cell survival. This has not been reported previously; however, this finding corroborates with later investigations indicating that AKT or protein kinase B is a critical signaling factor involved in insulin signaling, glucose metabolism, glycogen synthesis, and metabolic homeostasis. In fact, the absence of AKT activity is responsible for causing insulin resistance, obesity, and T2D mellitus. It has also been proven that microbial pigments impact pathways involved in glucose and lipid metabolism. This was evident from another investigation (El‐Sofany et al. 2025b), according to whom prodigiosin enhances insulin sensitivity and glucose balance through the regulation of metabolic targets linked to the insulin signaling pathway. There have also been several reports regarding the effect of pigments produced by Monascus on obesity, hyperlipidemia, and metabolism through insulin‐responsive pathways, namely, AKT and AMPK, by Ruan et al. (2026).

In cell experiments, the effectiveness of IND towards improving glucose metabolism in human spermatozoa, a high metabolic rate system, was assessed. In fact, the spermatozoa of humans require a vast amount of energy due to its high metabolic rate for motility, capacitation, and fertilization. The ATP used for this purpose comes from the mitochondria‐rich midpiece where many of the important signaling pathways related to energy homeostasis and cell survival are activated. AMPK works like a cellular energy sensor and is responsible for keeping the ATP homeostasis in times of energy consumption (Hurtado de Llera et al. 2013; Martin‐Hidalgo et al. 2018), while Akt is involved in sperm viability, motility, capacitation, and the resistance of sperm to oxidative stress by acting through the PI3K/Akt pathway (Zhao et al. 2016). Hence, in general, both AMPK and Akt must be activated considerably to secure proper energy metabolism, mitochondrial functioning, and fertilizing capacity of spermatozoa. IND showed an increase in glucose metabolism up to 133.4% at 20 µg/mL, with similar results as those of A‐769662 and SC79, and having activity even at high concentrations; therefore, it can be concluded that the molecule is less susceptible to toxic effects and more stable metabolically. This result is associated with the involvement of AMPK and AKT signal pathways in glucose metabolism improvement. IND activated AMPK up to 128.9%, with a dose‐dependent increase in AKT activity up to 121.4%. This result demonstrates improved glucose intake and homeostasis, as well as insulin signaling. Considering all the above facts, it can be concluded that IND is a potential natural drug for obesity and diabetes management. This study has shown that IND has a significant effect on glucose metabolism in human spermatozoa, a very metabolically active cell line known for high levels of mitochondrial activity, which can be explained by the fact that glucose is an important source of energy necessary for proper sperm motility, capacitation, and fertilization ability (Costa et al. 2023; Lacalle et al. 2025). The mechanism behind this may include AMPK pathway activation because AMPK is one of the most important regulators of energy balance that acts as a sensor and helps increase glucose metabolism in situations when there is a metabolic stress condition (Pang et al. 2018). Moreover, AMPK activation in sperm cells was associated with improvement in motility, mitochondrial activity enhancement, and higher fertilization ability (Li et al. 2023; Rahman et al. 2026). At the same time, the activation of AKT pathway indicates better insulin and glucose metabolism as AKT is responsible for energy metabolism regulation (Deng et al. 2021; Lee et al. 2025). This synergism between the activation of AMPK and AKT would be especially pertinent, since these two signaling pathways can be complementary in action in order to ensure optimal energy balance and germ cell survival. Lastly, the inhibitory effect of IND against pancreatic amylase and lipase activity may be seen as another factor pointing to its therapeutic potential regarding the improvement of metabolic function by virtue of AMPK activation. Despite the promising biological activities demonstrated by IND against α‐amylase, pancreatic lipase, AMPK, and AKT, some limitations should be noted:

1. In vivo studies are required to validate the metabolic effects of IND.
2. Further toxicity and safety assessments are needed.
3. Pharmacokinetic and bioavailability studies remain necessary.
4. The molecular mechanisms of AMPK and AKT modulation require further investigation.
5. Formulation optimization and large‐scale production should be explored for future applications.

## 5 Conclusion

This research shows that using optimized LB medium is beneficial for IND production compared to standard medium. IND was shown to be stable under different pH, temperature, and storage conditions, although it is sensitive to light. To our knowledge, this is the first report on IND being an inhibitor for α‐amylase and lipase while facilitating AMPK and AKT signaling pathway activation, which are the major metabolic regulators for glucose and lipids and associated with diabetes, obesity, and insulin resistance. The molecular docking and cellular studies confirmed the results and additionally indicated lower toxicity for IND than for A‐769662 and SC79 used in the experiment at high doses. Even though the results give us promising preliminary evidence regarding the potential of IND as a multifunctional natural pigment used in nutraceuticals and pharmaceuticals, further investigations are required before the introduction of IND for therapeutic purposes. Specifically, in vivo studies are needed to confirm metabolic properties, toxicity and safety tests must be carried out, pharmacokinetics and bioavailability must be established, and molecular mechanisms responsible for AMPK and AKT activation must be determined.

## Author Contributions

**Tahani Ali Asseri:** conceptualization, investigation, funding acquisition, writing – original draft, writing – review and editing, visualization, validation, methodology, software, formal analysis, project administration, resources, supervision, data curation.

## Ethics Statement

The author has nothing to report.

## Conflicts of Interest

The author declares no conflicts of interest.

## Data Availability

The data that support the findings of this study are available on request from the corresponding author. The data are not publicly available due to privacy or ethical restrictions.
